# To Hop or Not to Hop: Unveiling Different Modes of Ion Transport in Solid Polymer Electrolytes through Molecular Dynamics Simulations

**DOI:** 10.1021/acsapm.4c03724

**Published:** 2025-04-15

**Authors:** Harish Gudla, Anne Hockmann, Daniel Brandell, Jonas Mindemark

**Affiliations:** † Department of Chemistry - Ångström Laboratory, Uppsala University, Box 538, SE-751 21 Uppsala, Sweden; ‡ Institute of Physical Chemistry, University of Münster, Corrensstrasse 28/30, 48149 Münster, Germany

**Keywords:** ion-transport mechanisms, solid polymer electrolytes, molecular dynamics simulations, ion coordination, transport modes

## Abstract

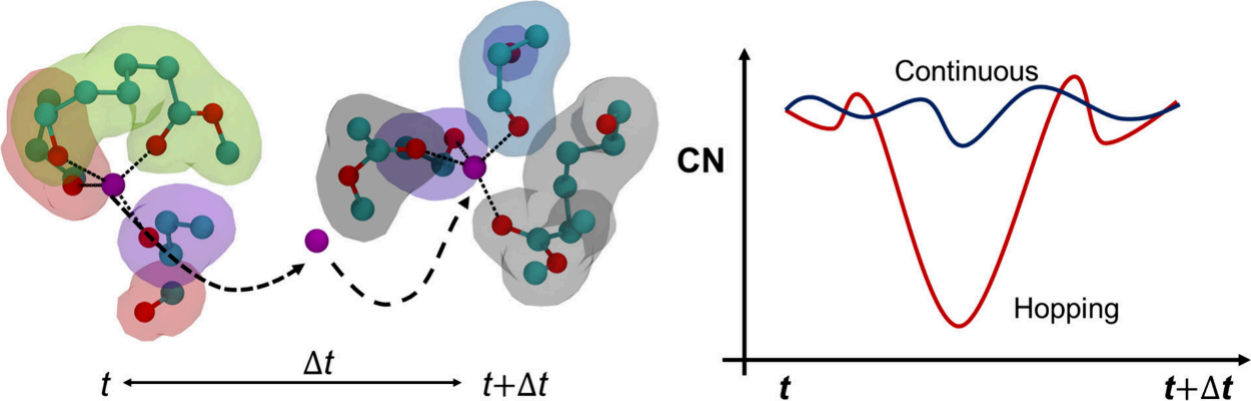

Although the basic modes of ion transport in solid polymer electrolytes (SPEs) are already classified and well-described, their distribution in typical polymer electrolytes is not clear and neither are the effects on the distribution by different degrees of ion–ion and ion–polymer interactions. Here, the ion-transport mechanisms in poly(ethylene oxide) are studied along with poly(ε-caprolactone) at different molecular weights and LiTFSI salt concentrations using molecular dynamics simulations. Through tracking of the cation coordination changes, three transport mechanisms are categorized, i.e., ion hopping, continuous motion (successive exchange of the coordination sphere), and vehicular transport. The observed dominant transport mechanism is in all cases continuous motion, which changes from polymer-mediated to anion-mediated with increasing salt concentration, while polymer-mediated vehicular transport is not observed to be a major contributor to cation transport. In both systems, ion hopping is also essentially absent, as can be expected in systems with strong ion–polymer interactions. The results illustrate how the usual description of ion transport in polymer electrolytes as coupled to segmental motions is too simplistic to catch the full essence of the ion-transport phenomena, whereas the frequently used notion of “ion hopping” in the majority of cases is incorrect for SPEs.

## Introduction

Solid polymer electrolytes (SPEs) are often hailed as safer and more structurally and mechanically versatile alternatives to the flammable liquid electrolytes currently in use in commercial lithium-ion batteries, with the main drawback being limited ionic conductivity.^1−4^ Attaining critical advancements in this area requires a thorough understanding of the mechanisms of ion transport in solid polymer matrixes. Ion transport in polymer electrolytes has been explored for the past few decades^5−8^ with the main focus being on systems based on poly(ethylene oxide) (PEO) due to its excellent lithium solvation properties and comparatively good ionic conductivity.^4,9^ Extensive studies on PEO systems have highlighted the dependence of ion transport on the segmental mobility of the polymer, describing the transport of solvated cations as coupled to the segmental motions of the polymer chains.^10−12^ This is, however, a somewhat simplistic view that may not necessarily translate to a wider variety of polymer hosts. Instead, a more diverse mechanistic description should facilitate a more constructive discussion. We can find a good starting point in the classification by Son and Wang,^5^ distinguishing three main modes of ion transport based on the type of change in the local coordination shell: (i) the continuous mode, where the ion moves by successive exchanges of solvating groups; (ii) hopping between solvation sites; (iii) ion–polymer codiffusion—in a similar fashion to the vehicular transport in liquid electrolytes.

It is easy to envision the movement of cations as hopping or jumping along or between polymer chains, and indeed such descriptions can commonly be found in the literature.^10−14^ There exists, however, little evidence that this is a realistic picture of what really occurs in an SPE system. Moreover, the relative proportions of the different transport modes in terms of hopping and alternative mechanisms are often not quantified, even in theoretical works. The application of emerging methods such as metadynamics^15^ and correlation functions^16^ may provide important insights into the free energy landscape as a function of different coordination environments, and the connection between electrolyte component interactions and observed transport phenomena, respectively. However, neither of these methods ultimately provides information on the distribution of actual transport events, for which an analysis of individual events is instead needed.

In an amorphous polymer matrix above the glass transition, the chains are dynamic and there are no fixed coordination sites for hopping between. Considering also that a proper “hop” entails exchanging the full coordination shell in a single event as in ceramic electrolytes; the considerable coordination energies in not least PEO systems should make this event rare.^17^ It can thus be questioned whether it is then at all relevant to talk about hopping in dynamic SPE systems. On the other hand, a hopping mechanism has recently been shown to be active in solutions of Li salts in glycerol,^18^ indicating that such a mechanism can indeed be an active mode of transport also in dynamic systems.

In addition, the aforementioned classification model only reflects ion transport as mediated through coordination between the cation and polymer. This may be relevant in SPEs at (very) low salt concentrations where direct cation–anion interactions are minimal, but at more realistic salt concentrations these interactions cannot as easily be neglected, and balanced interactions between the cation, anion, and polymer are required.^19^ This necessitates amendments of the mechanistic descriptions to take all interactions into consideration when unveiling the transport mechanisms and becomes particularly relevant when approaching the polymer-in-salt electrolyte (PISE) regime.^20,21^ The PISE category of SPE materials often displays high decoupling of ion transport from polymer dynamics where ion transport is suggested to follow the continuous exchange of anions, like in ionic liquids.^22^

One important reason why a more general model is needed is the ability to apply it to a wider variety of host materials. PEO-based systems are generally limited by low cation transference numbers at high molecular weights.^23,24^ Polymers based on carbonates or esters, on the other hand, are promising alternatives due to their weak Li^+^ complexation, resulting in high transference numbers.^1,25^ In recent years, systems based on biodegradable poly(ε-caprolactone) (PCL) have received growing interest because they have thermal properties similar to those of PEO but show high transference numbers.^26−28^ The different ion coordination strengths and cation transference numbers in these systems should reasonably be reflected also in the ion-transport mechanism. A detailed study of the differences between PEO and PCL could provide further insights into the transport mechanisms and their dependence on coordination properties.

In this work, we apply a quantitative model based on ion coordination that could be transferred to any SPE over a wide range of molecular weights and salt concentrations in order to distinguish what transport mechanisms are actively contributing to cation movement in PEO:LiTFSI and PCL:LiTFSI electrolytes. This model is applied to a range of both molecular weights and salt concentrations while observing the differences not only between vehicular, continuous, and hopping modes of ion transport but also between mediation of these modes by either the polymer chains or the negatively charged counterions for a comprehensive description of ion transport in this diverse range of electrolyte systems. The paper is organized so that first the criteria for defining different ion-transport mechanisms in the molecular dynamics simulations are evaluated. Thereafter, these criteria are employed to analyze ion transport in PEO:LiTFSI and PCL:LiTFSI as representatives of SPEs with different ion coordination characteristics.

## Methods

### Molecular Dynamics (MD) Simulations

MD simulations were performed for both polymers at different molecular weights (chain lengths) and LiTFSI salt concentrations; details are shown in Table S1. The simulation boxes for all systems contain a total of 1000 monomer units, and the number of Li and TFSI ions are varied, corresponding to the concentration ratio *r* = [Li^+^]/[monomer]. General AMBER force field (GAFF) parameters were used to describe the interactions between the polymer chains and ions. The particle charge of the ions was scaled by a factor of 0.75 to get better agreement with experiments.^19,29^ The initial packing of the simulation box was generated using *PACKMOL* (version 17.333).^30^ After minimization of the total energy of the system using the steepest descent algorithm, MD simulations using the software package *GROMACS* (version 2021)^31^ were performed to equilibrate the system. The leapfrog algorithm was used with a time step of 1 fs for all MD simulations. The first simulation was conducted in an NVT (constant number of particles, volume, and temperature) ensemble at 400 K for 5 ns using a modified Berendsen thermostat to equilibrate the temperature.^32^ The second equilibration run was performed in an NPT (constant number of particles, pressure, and temperature) ensemble for 10 ns using a Parrinelllo–Rahman barostat at 1 bar to equilibrate the density.^33^ During this run, the temperature was ramped from 400 to 1000 K and back to 400 K to ensure good equilibration and relaxation of the polymer chains. After that, a second NPT run of 10 ns at the desired temperature was performed before the final NPT production run with a simulation time of 500–800 ns at 440 K was started. The energies and trajectories were saved every 0.1 and 5 ps, respectively. For the categorization of transport mechanisms, oxygen index number lists and total coordination number (CN) lists were sampled every 25 ps. To determine the glass transition temperature (*T*_g_) of the simulated systems, another NPT run was performed where the temperature was decreased stepwise from 540 to 140 K with a step size of 20 K. Each temperature was simulated for 4 ns, and the average density of the system was calculated from the last 2 ns.

### Pulsed Field Gradient (PFG) NMR

Diffusion coefficients for the PCL:LiTFSI electrolytes were measured at 333 K on a Bruker Avance III HD 400 MHz NMR spectrometer equipped with a Bruker Diff50 gradient probe head (field gradient strength of up to 30 T m^–1^). For all investigated nuclei and low molecular weights, the observation time Δ was set to 300–500 ms and the gradient pulse length δ to 1–2 ms, with the higher values being used for ^7^Li for higher molecular weights. The maximal gradient strength *g*_max_ varied between 999 and 2874 G cm^–1^ depending on the sample and measured nucleus. PCL with different molecular weights was synthesized by ring-opening polymerization of ε-caprolactone, analogous to the method described for the synthesis of poly(trimethylene carbonate), and polymer electrolytes were prepared by solution casting from anhydrous acetonitrile, following a method analogous to that of Eriksson et al.^34^

## Results and Discussion

### Defining Criteria for Distinguishing Different Modes of Ion Transport

With similar thermal properties (Figure S1) but distinct differences in ion coordination, PEO and PCL were used as model polymers to cover a wide variety of relevant conduction mechanisms. For the same purpose, three different molecular weights were utilized (Table S1), with the highest molecular weight being definitely above the entanglement limit. Also, three different salt concentrations (*x* = [Li^+^]/[monomer]) of 0.08, 0.7, and 1.0 were used to cover both the conventional salt-in-polymer regime (*x* = 0.08) and the PISE domain (*x* = 1.0). The notation used to represent a particular system is Polymer_*n*_:S_*x*_; for example, PEO_25_:S_0.7_ represents a PEO:LiTFSI system with a polymer chain length equivalent to 25 repeating units, *M*_n_ = 1119.33 g mol^–1^, and salt concentration *x* = 0.7.

**Figure 1:**
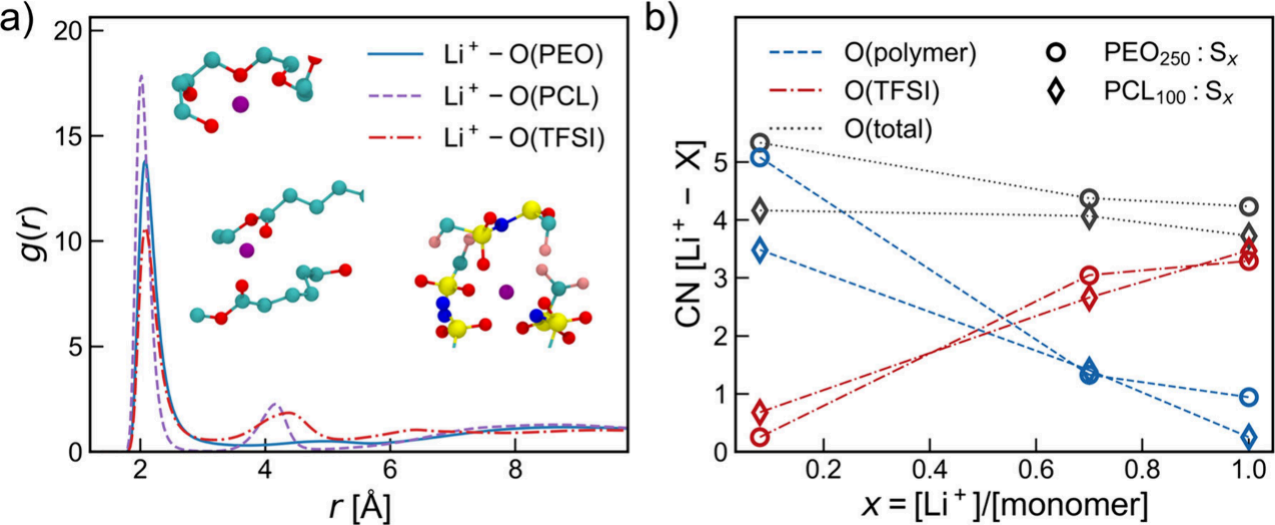
(a) Radial distribution functions *g*(*r*) for Li^+^ with O atoms from the polymer backbone for PEO_250_:S_0.7_ and PCL_100_:S_0.7_ systems and with O atoms from TFSI for the PEO_250_:S_0.7_ system along with their representative local coordination. Color code: Li^+^, purple; O, red; C, cyan; N, blue; S, yellow; F, pink. (b) CNs of Li^+^–O(polymer), Li^+^–O(TFSI), and Li^+^–O(total) in the first coordination shell [first minimum in *g*(*r*)] as a function of the salt concentration for PEO_250_ and PCL_100_ systems. Here, Polymer_*n*_:S_*x*_ represents a particular system with polymer chain length *n* and salt concentration *x*.

The local solvation environment of Li^+^ can be estimated from radial distribution functions *g*(*r*), and the peaks in *g*(*r*) depict the presence of specific targeted atomic species (X) at a distance *r* from Li^+^. In Figure 1a, the *g*(*r*) of Li^+^–O(X) for an intermediate molecular weight and *x* = 0.7 (PEO_25_:S_0.7_ and PCL_24_:S_0.7_) are shown, and the *g*(*r*) of all systems can be found in Figure S2. From *g*(*r*) of Li^+^–O(polymer), some differences between Li^+^ coordination in the two polymers can be observed. The first maxima of *g*(*r*) for Li^+^–O(PCL) and Li^+^–O(PEO) are located at 2.0 and 2.1 Å, respectively. These values are in good agreement with previous experimental^35,36^ and simulation studies.^10,28^ The second peak in *g*(*r*) of around 4 Å observed only for Li^+^–O(PCL) can be attributed to the alkoxy group in the PCL chain, which does not directly coordinate to Li^+^ but is geometrically related to the coordinating carbonyl oxygen and therefore follows at a fixed distance. The *g*(*r*) of Li^+^–O(TFSI), here exemplified for the PEO_25_:S_0.7_ system, shows two peaks, one at 2.1 Å that accounts for the coordination of Li^+^ to O atoms and a broad peak at 4.2 Å for the O atoms of the same or neighboring TFSI anions. This, along with the second peak in *g*(*r*) of Li^+^–N(TFSI) in Figure S2, especially at higher salt concentrations, depicts the presence of ion triplets or higher-order ionic clusters. A change in these peak heights can be observed from *g*(*r*) in Figure S2 with an increase in the salt concentration or molecular weight, which corresponds to changes in the coordination environments.

To understand these changes, CNs were derived from integrating the respective *g*(*r*) up to a cutoff distance of 3 Å, i.e., the first coordination shell, and plotted in Figure 1b. With an increase in the salt concentration, the Li^+^ environment changes from predominantly coordination by the polymer (three to five O atoms in the polymer) to about three O atoms of TFSI and not more than one O atom from the polymer. This trend can be observed for both polymer electrolyte systems and irrespective of the molecular weight (Figure S2c,d). However, the total CN of Li^+^–O in PEO:LiTFSI systems is higher than that in PCL:LiTFSI systems due to a lower CN by O in the polymer, possibly because the coordinating O atoms are less accessible in PCL due to steric effects, which could also be observed from snapshots in Figure 1a.

A more fundamental discussion of the ion-transport metrics requires a more in-depth and detailed description of the active ion-transport mechanisms. While *g*(*r*) and CN only provide the static picture of local coordination environments, the ion-transport mechanisms can be estimated by analyzing the evolution of these environments. In essence, we can distinguish three main types of transport mechanisms by the rate of ligand exchange in their coordination shell in comparison to the rate of ion movement in the system. If these occur on the same time scale, the transport is continuous (coupled to the chain dynamics of the system). If the ligand exchange is much slower than the rate of ion movement, the transport occurs through codiffusion (vehicular transport). Finally, hopping entails ligand exchange that is (simultaneous and) much faster than the ion movement. Accordingly, to capture these nuances in a quantitative assessment, the changes of the first coordination shell of Li^+^ were analyzed with respect to time. The goal of this evaluation is to compare the coordination environments of each Li^+^ at a time *t* and *t* + Δ*t*, where Δ*t* is the time step. According to the observed changes in the coordination, each event is assigned to a particular transport mechanism occurring at this time step. For an event to be classified as ion hopping, the total CN needs to be ≤1 at some intermediate point, reflecting a (near) total change in the coordination environment. In a similar fashion, vehicular transport is defined as an identical coordination before and after the transport event, while allowing a small fluctuation in the CN of up to 1 during the intermediate time. Everything between these extremes is considered to be continuous transport. By averaging over all cations in the system and at all time steps, a probability of each transport mode can be obtained.

The division into these three transport modes, however, risks understating the influence of the anions, particularly at high salt concentrations where the cations are more coordinated by anions than by polymer chains (Figure 1b). To capture this dimension and further distinguish between polymer–ion and ion–ion interactions, these mechanisms were further categorized into polymer- and anion-mediated transport depending on the number of O atoms from each species at *t* and *t* + Δ*t*. If CN[Li^+^–O(polymer)] ≥ CN[Li^+^–O(TFSI)], then the event is considered to be polymer-mediated transport. The method used in this work to observe the changes in coordination is based on previous MD studies on polymer electrolytes^14^ and polymerized ionic liquids^37,38^ but has here been extended to include coordination changes of both anions and polymers, together with changes in the total CN. The detailed definitions and procedure to characterize these ion-transport mechanisms are described in the Supporting Information (see section S4 and Figure S3, with representative snapshots of the vehicular and continuous modes shown in Figure S4).

**Figure 2:**
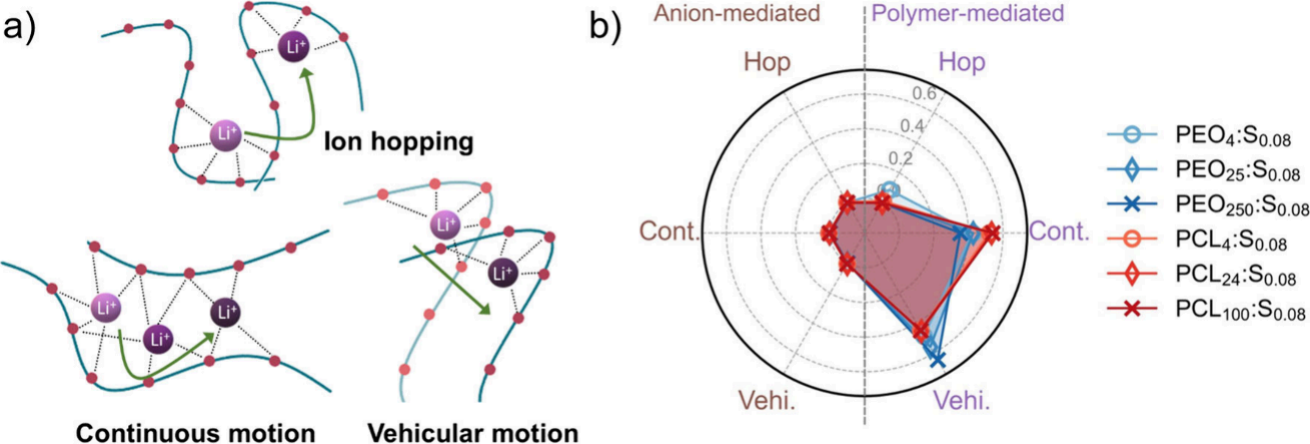
(a) Schematic description of polymer-mediated transport categorized based on changes in the cation coordination shell. (b) Probability of the type of ion-transport mechanisms for both the polymers at different molecular weights with salt concentration *x* = 0.08, i.e., PEO_*n*_:S_0.08_ and PCL_*n*_:S_0.08_. Here, Δ*t* = 0.1 ns. The mechanisms are further categorized into polymer-mediated (purple; right) and anion-mediated (brown; left) ion-transport modes.

**Figure 3:**
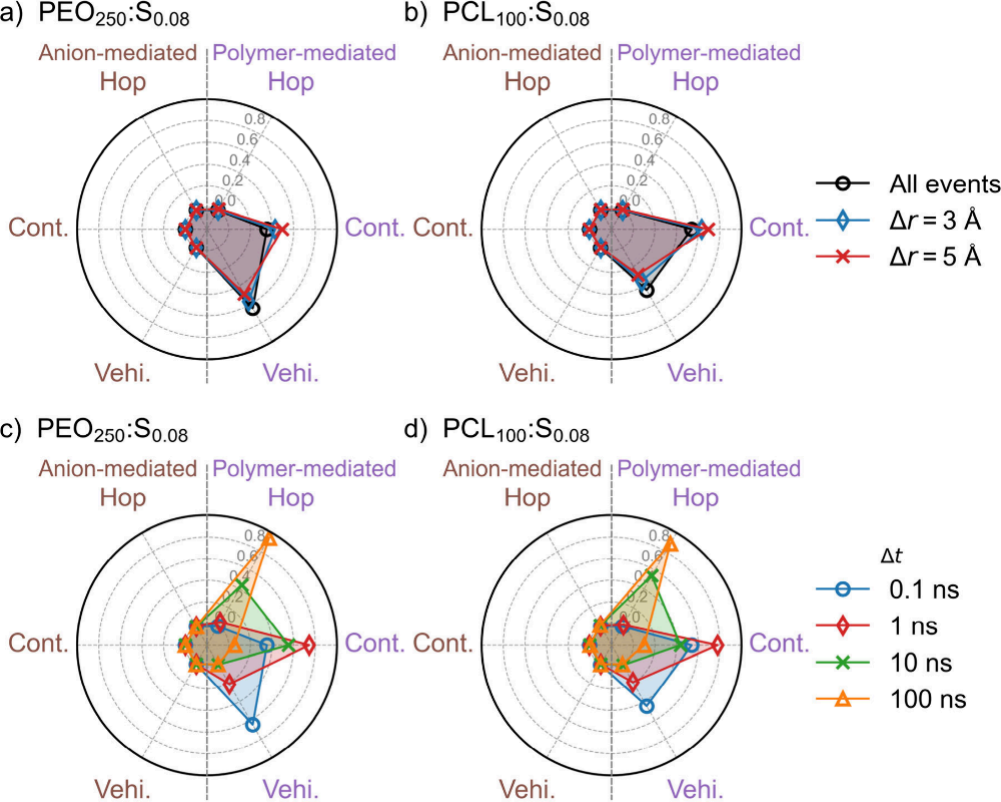
Probability of the type of ion-transport mechanisms for the systems PEO_250_:S_0.08_ (a) and PCL_100_S_0.08_ (b) where either all (essentially Δ*r* = 0) or effective events with Δ*r* = 3 or 5 Å are considered, where the time step (Δ*t*) is 0.1 ns. Probability of the type of ion-transport mechanisms for the systems PEO_250_:S_0.08_ (c) and PCL_100_S_0.08_ (d) at different time steps (Δ*t*) with Δ*r* = 3 Å.

The radar plot in Figure 2b shows the probability of all transport mechanisms with a time step of Δ*t* = 0.1 ns. For both polymer systems, polymer-mediated transport is predominant at the low salt concentrations (*x* = 0.08) because Li^+^ is primarily coordinated to the polymer (Figure 1b) and there seems to be similar contributions from continuous and vehicular motion. These observed trends were unaffected with increased molecular weight for PCL systems, but for PEO, the probability of continuous motion was reduced, and the vehicular motion was increased. This increase in the probability of vehicular motion with increased molecular weight for PEO:LiTFSI might seem highly counterintuitive because the diffusion of high-molecular-weight polymer chains should be negligible and the vehicular transport mode should therefore be diminished. The answer to this lies in what constitutes an effective ion-transport event. The classification in Figure 2b is based on changes in Li^+^ coordination but does not distinguish whether the ion has actually moved an appreciable distance. To address this, a distance cutoff Δ*r* is necessary, defined as the distance traveled by Li^+^ during Δ*t*. As exemplified in Figure 3a,b, one can observe an increase in vehicular motion when Δ*r* is decreased, whereas there is a decrease in continuous motion. Considering that these data are for a high-molecular-weight system (above the entanglement limit), vehicular codiffusion is unlikely, given the very limited diffusion of large polymer chains. The vehicular mode, therefore, becomes overestimated at low values of Δ*r* because even small fluctuations where Li^+^ neither moves significantly nor changes its coordination are counted as vehicular events. With a small Δ*t*, such events will be highly frequent in comparison to the other transport modes, which highlights the local dynamics of the system as consisting of a series of local fluctuations within a fixed coordination environment, mimicking vehicular transport, followed by more productive transport events. The large proportion of vehicular events suggests that such local vehicular fluctuations tend to dominate the ion dynamics and that the other transport modes, which contribute more to long-range ion transport, are comparatively rare.

From here on, the transport events are considered effective only if Li^+^ is dynamic, i.e., if it has traveled a minimum cutoff distance Δ*r*. However, the percentage of effective events decreases exponentially with an increase in Δ*r*, as shown in Figure S5a. In this work, Δ*r* = 3 Å was chosen such that any event where Li^+^ has traveled out of its first coordination shell is considered an effective event. As can be seen in Figure S5a, at this cutoff distance, for low salt concentration systems, 40–70% of the events were effective and, for higher salt concentration, at least 10% of effective events were observed.

As for the size of the time step Δ*t*, as shown in Figure 3c, at Δ*t* = 0.1 ns, vehicular and continuous motion can be observed. With an increase in Δ*t* to 1 ns, the ratio of vehicular to continuous motion is reduced. This indicates that vehicular transport is predominant only at small applied time steps. With a further increase in Δ*t*, the continuous motion is gradually shifted to ion hopping. So, at higher molecular weights, provided a long enough time interval, the ion-hopping mechanism is observed to dominate over all other transports. This is, however, a mirage, as indicated by the gradual shift from vehicular over continuous motion to hopping; if the time step is sufficiently large, a single hopping event at any point during the time period will classify the event as a hop even if some other transport mode is responsible for the majority of the distance traveled. This leads to a sensitivity of the classification of the transport modes to the length of the time step that needs to be addressed.

**Figure 4:**
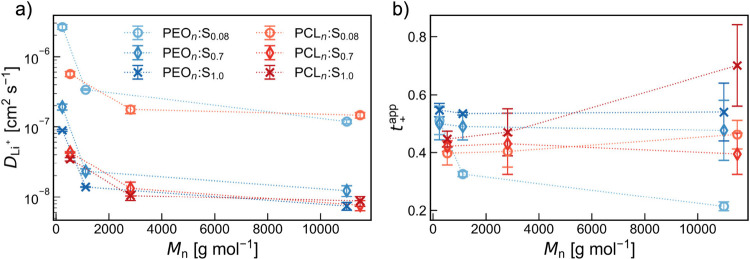
(a) Li^+^ self-diffusion coefficients (*D*_Li^+^_) and (b) apparent cation transference number (*t*_+_^app^) for both polymers PEO and PCL at different salt concentrations as a function of molecular weight (*M*_n_).

Calculating the average residence times of Li^+^–O(polymer) (τ_Li–O(polymer)_) and Li^+^–O(TFSI) (τ_Li–O(TFSI)_) gives an insight into the time scale of Li^+^ solvation dynamics, which helps in establishing a carefully considered time step criterion. The details of the computation of τ_Li–O(X)_ are given in section S5. From Figure S6, the residence times increase from low to intermediate molecular weights and appear to stabilize at high molecular weights. Similar trends can be seen with an increase in the salt concentration. One can also observe that these trends are inverse to the trends in Li-ion diffusion in Figure 4a. This inverse relationship correlates well with the Walden rule or Stokes–Einstein relationship that is generally seen for organic electrolytes, polymerized ionic liquids, and polymer electrolytes.^38−40^ The residence time of Li^+^ with polymer chains (Figure S6a) is always 1–2 orders of magnitude higher than the residence time of Li^+^ with anions (Figure S6b). This suggests that Li^+^ solvation is more dynamic when it coordinates with an anion. Because the residence times were averaged over all coordination environments, a time step Δ*t* below these τ_Li–O(X)_ values could be used to fully cover the full range of dynamics in all environments. A standard time step of Δ*t* = 1 ns can therefore be considered suitable for all systems; at this time step, the continuous transport mode is dominating, and at least 50% of the events are effective with Δ*r* = 3 Å (Figure S5b).

### Quantitative Assessment of Ion-Transport Modes

With the mechanistic criteria established, we can start to investigate how changes in the ion-transport mechanism affect the mode of ion transport in the studied systems. To compare the ion dynamics in the polymer electrolyte systems, the self-diffusion coefficients of Li^+^ (*D*_Li^+^_) were calculated from mean-square displacements and are shown in Figure 4a. The *D*_Li^+^_ values for PEO_*n*_:S_0.08_ were in good agreement with previous experimental^41^ and simulation studies in terms of both trends and absolute values, thereby validating the force field for this system.^14^ From Figure 4a, it can be observed that *D*_Li^+^_ decreases with an increase in the molecular weight for both polymer systems, which can be attributed to a decrease in the polymer segmental mobility, as evidenced by the increase in *T*_g_ (Table S1). With an increase in the salt concentration from *x* = 0.08 to 0.7, *D*_Li^+^_ decreases by 1 order of magnitude, with similar values for *D*_Li^+^_ seen also for *x* = 1.0. With a decrease in the chain mobility as a result of physical cross-links that appear when salt is added to the system, this could be expected if assuming a traditional continuous transport mechanism coupled to segmental motions of the polymer host. However, changes in transport modes would also affect these values.

**Figure 5:**
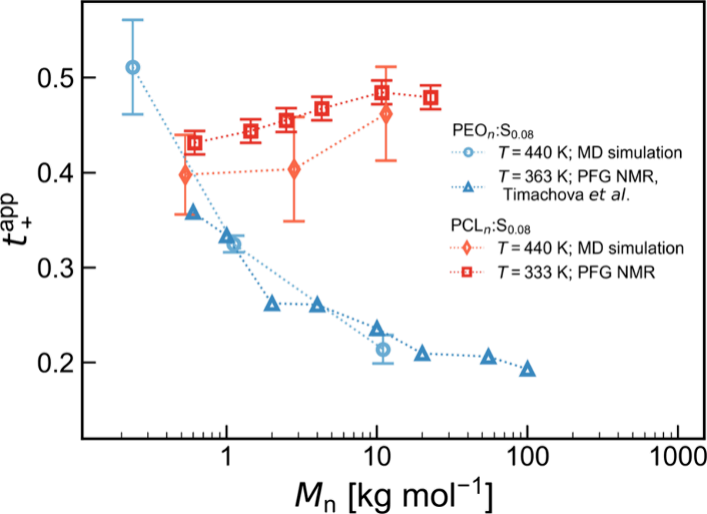
Comparison of *t*_+_^app^ from MD simulations and PFG NMR. Experimental data for PEO:LiTFSI were taken from Timachova et al.^41^

To further characterize the ion transport, apparent cation transference numbers (*t*_+_^app^) were calculated as the ratio of *D*_Li^+^_/(*D*_Li^+^_ + *D*_N(TFSI)_) and are shown in Figure 4b. At the lowest salt concentration, a decrease in *t*_+_^app^ as a function of the molecular weight is observed for PEO. This trend closely mirrors available experimental data obtained through both PFG NMR and electrochemical methods (Figure 5).^13,41,42^ The coordination data in Figure S2 suggest that the local ion coordination does not change substantially with molecular weight, which would indicate that the change in *t*_+_^app^ is due to a switch in ion-transport mechanism. However, *g*(*r*) does not capture the full picture of ion coordination. It has recently been shown that the ion coordination strength is dependent on the molecular weight,^42^ correlating with the change in the transference number and suggesting that the transport mechanism remains continuous throughout. The same decrease in *t*_+_^app^ with molecular weight is not seen for PCL. Again, the trend mirrors that seen in data from PFG NMR (Figure 5). At high molecular weight, the values are higher for PCL than for PEO, which has been attributed to the lower coordination strength of Li^+^ with the carbonyl oxygens of PCL than with the ether oxygens of PEO.^25^

**Figure 6:**
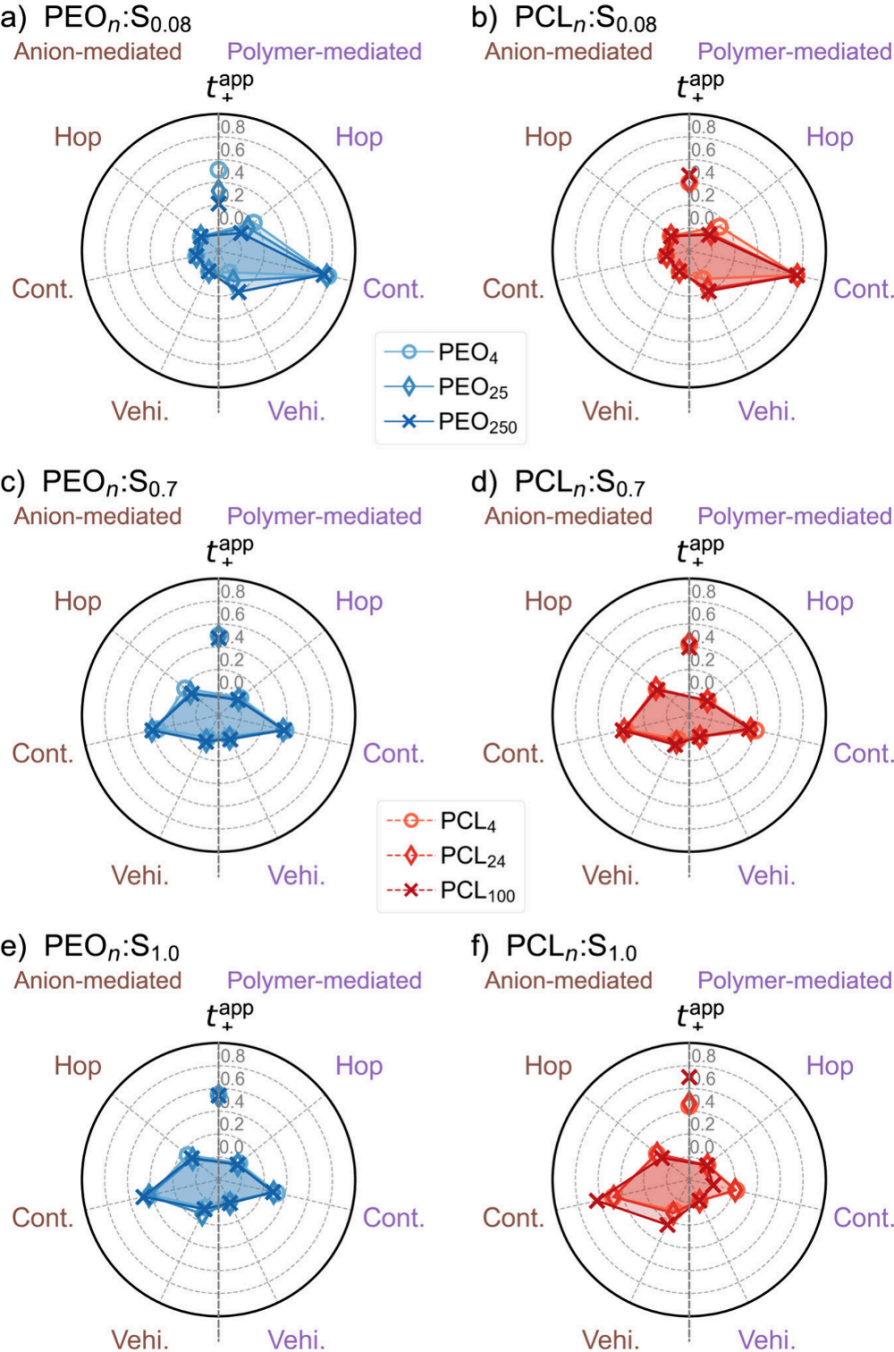
Probability of the type of ion-transport mechanisms and Li^+^ transference number as a function of molecular weight for both PEO (a, c, and e) and PCL (b, d, and f) at different salt concentrations with time step Δ*t* = 1 ns and effective distance criterion Δ*r* = 3 Å. The mechanisms are further categorized into polymer-mediated (purple; right) and anion-mediated (brown; left) ion transport.

Using the established distance and time criteria, i.e., Δ*r* = 3 Å and Δ*t* = 1 ns, the probability of each transport mechanism was determined and can be seen plotted in Figure 6 for all systems. In addition, the cation transference numbers (*t*_+_^app^) are also plotted alongside the probabilities to help provide more insights into the trends observed in Figure 4b. At the low salt concentration (*x* = 0.08), the ion transport is predominantly polymer-mediated for both PEO and PCL systems (Figure 6a,b), with the dominant transport mode being continuous. The difference between PEO and PCL systems can be observed from the percentage of anion dynamics involved during this continuous motion, as shown in Figure S5c. For PCL, more anion involvement (80%) is seen, twice that of PEO. The higher anion assistance in PCL can likely be attributed to the higher anion coordination (CN [Li^+^–O(TFSI)] in Figure 1b) as a result of weaker Li^+^ binding to PCL than to PEO. At this concentration, the decrease seen in *t*_+_^app^ for PEO as a function of *M*_n_ is correlated with an increase in the anion dynamics during the continuous transport mode, as was also recently suggested for similar systems based on experimental data.^25,42^

In all cases, even at the lowest molecular weight, the contribution of the vehicular transport mode was found to be minimal. We note that this is in contrast to Borodin et al., who reported a significant contribution from vehicular transport at both high and low (*n* = 6) molecular weight.^43^ However, they treated each polymer chain as a single solvent molecule, thereby ignoring intrachain continuous movement as well as the effects from coordination of an ion by several chains simultaneously. With decreasing molecular weight, one would expect an increasing incidence of vehicular transport. However, as seen in Figure 6, in some systems, the opposite is seen. These events are likely comparatively short-distance fluctuations of the entire coordination structure that do not directly contribute to the global conductivity but that may move the coordinated Li^+^ ion into a position where it can be transferred through continuous transport modes to a new coordination environment. This type of dynamics characterized by a high frequency of local fluctuations in a vehicular fashion, sometimes interrupted by changes in coordination that enable an ion to more efficiently move longer distances, has previously been described as a mechanism of “cage trapping and hopping” for a PEO:LiTFSI_0.1_ system.^44^

At higher salt concentrations (*x* = 0.7 and 1.0) for both PEO (Figure 6c,e) and PCL (Figure 6d,f), it is possible to observe a shift from polymer- to anion-mediated transport. This shift is perfectly reasonable given the high salt concentration and, therefore, a high number of anions in the coordination environment of the Li^+^ ions (Figure 1b), with the dominant transport mechanism for these systems being the anion-mediated continuous motion. At the salt concentration *x* = 0.7, the transport mechanism seems to be independent of *M*_n_, which could suggest that cation transport is partially decoupled from the polymer dynamics for both PEO and PCL systems.

When the salt concentration is further increased to *x* = 1.0, the distribution of transport modes is similar for the PEO systems compared to *x* = 0.7 (Figure 6c,e). However, an increase of 15–30% in the total anion-mediated mode can be noted, which is correlated with an increase in the cation transference number at the highest salt concentration. In the PCL systems at the highest salt concentration (Figure 6f), anion-mediated continuous motion is clearly the dominant transport mechanism, followed by anion-mediated hopping (hopping within or between ionic clusters). At the highest *M*_n_, polymer-mediated transport is essentially absent, suggesting that ion transport is fully decoupled from the polymer segmental motion, forming a PISE material. In the PISE regime, ion transport is reported^45^ to be similar to ion transport in ionic liquids, which agrees well with the data in Figure 6f. The formation of a highly conductive PISE regime in PCL compared to PEO is likely associated with the weaker ion coordination in PCL, allowing ion transport to be fully decoupled and anion-mediated. This is apparently not possible for the stronger-coordinating PEO, leading to a stronger-bound and immobilized cation.

For the PCL system with *x* = 1.0, the apparent transference number also drastically increases from 0.4 to 0.7 with an increase in the molecular weight, which is not observed in the other systems. This change in ion-transport characteristics aligns with the formation of a PISE phase at high salt concentrations in the high-molecular-weight PCL system, whereas no such clear indication is seen for PEO. In particular, the molecular weight dependence is notable, with a steep increase in the transference number and the incidence of anion-mediated continuous ion transport indicating PISE formation only being observed for the highest molecular weight. Nevertheless, a general high *t*_+_^app^ at high salt concentrations associated with a high degree of anion-mediated continuous motion is also noted for PEO (Figure S8b). This correlation may be related to the different Li^+^ residence times with polymer and anion, suggesting that ion transport is coupled to both the polymer and anion motion.

As discussed before, these transport mechanisms are highly dependent on the applied Δ*r* and Δ*t*, so these probabilities at different Δ*r* and Δ*t* values are shown in Figure S5. However, the main observations are not affected by these changes. There are also only minor effects of changing the end groups from hydroxyl (as discussed so far) to methoxy; details can be found in section S8.

Importantly, the probability of polymer-mediated ion hopping at higher molecular weights was observed to be very low or zero for most of the systems. This shows that, even though ion hopping may constitute highly effective transport events, they are rarely observed and do not influence the overall ionic transport. It is therefore not reasonable to describe hopping as a productive ion-transport mechanism in most polymer electrolyte systems, at least not those based on PEO or PCL. To increase the probability of hopping, the energy barrier associated with a hop would need to be reduced by employing much weaker-coordinating polymer hosts. Another option would be to reduce the likelihood of the other transport modes by slowing down the chain and anion dynamics to a point where the continuous and vehicular modes become ineffective.

## Conclusions

In this work, all-atom MD simulations were performed for PEO:LiTFSI and PCL:LiTFSI at different molecular weights and salt concentrations to study the ion-transport mechanisms, distinguishing between hopping, vehicular, and continuous motion. These mechanisms were further subdivided by considering both polymer- and anion-mediated motion. By analysis of the time evolution of the local coordination environment surrounding each cation, a quantitative description was obtained.

With increased salt concentrations, the dominant transport mechanism changes from polymer-mediated continuous motion to anion-mediated continuous motion. At very high salt concentrations, corresponding to PISE systems, ion transport becomes decoupled from the polymer segmental motion for PCL systems due to their weak Li^+^ binding. The shift to anion-mediated continuous motion furthermore appears to be correlated with increasing *t*_+_^app^ irrespective of polymer and salt concentrations. The anion–cation coordination is thus of high importance for key transport properties in polymer electrolytes.

Interestingly, there is a very limited degree of ion hopping in these SPE systems, in particular between polymer chains. For the relevant time step and length-scale criteria, hopping between polymer chains is only observed for low molecular weights and salt concentrations, whereas anion-mediated hopping (hopping within or between ionic clusters) is observed at high molecular weights and high salt concentrations. This is in stark contrast to much of the scientific literature on SPEs, which often discusses ion transport in terms of “hopping” or “jumping”, although not always defining the mechanism properly. As shown here, however, such terms are unsuitable to describe cation transport in dynamic, amorphous polymer matrixes, where the constant rearrangement of chains and coordinating groups makes different continuous modes of transport much more probable.

## Supporting Information Available

Simulation details, glass transition temperatures, radial distribution and CN functions, definitions of ion-transport mechanisms, mean residence time calculations, mean-square displacements, and additional data on the probabilities of transport mechanisms and end-group dependencies (PDF)

## References

[ref1] Mindemark J.; Lacey M. J.; Bowden T.; Brandell D. Beyond PEO—Alternative host materials for Li+-conducting solid polymer electrolytes. Prog. Polym. Sci. 2018, 81, 114–143. 10.1016/j.progpolymsci.2017.12.004.

[ref2] Li Q.; Chen J.; Fan L.; Kong X.; Lu Y. Progress in electrolytes for rechargeable Li-based batteries and beyond. Green Energy Environ. 2016, 1, 18–42. 10.1016/j.gee.2016.04.006.

[ref3] Yang H.; Wu N. Ionic conductivity and ion transport mechanisms of solid-state lithium-ion battery electrolytes: A review. Energy Sci. Eng. 2022, 10, 1643–1671. 10.1002/ese3.1163.

[ref4] Xue Z.; He D.; Xie X. Poly(ethylene oxide)-based electrolytes for lithium-ion batteries. J. Mater. Chem. A 2015, 3, 19218–19253. 10.1039/C5TA03471J.

[ref5] Son C. Y.; Wang Z.-G. Ion transport in small-molecule and polymer electrolytes. J. Chem. Phys. 2020, 153, 100903 10.1063/5.0016163. 32933299

[ref6] Mogurampelly S.; Borodin O.; Ganesan V. Computer Simulations of Ion Transport in Polymer Electrolyte Membranes. Annu. Rev. Chem. Biomol. Eng. 2016, 7, 349–371. 10.1146/annurev-chembioeng-080615-034655. 27070764

[ref7] Brandell D.; Mindemark J.; Hernández G. Polymer-Based Solid State Batteries; De Gruyter: Berlin/Boston, 2021.

[ref8] Choo Y.; Halat D. M.; Villaluenga I.; Timachova K.; Balsara N. P. Diffusion and migration in polymer electrolytes. Prog. Polym. Sci. 2020, 103, 101220 10.1016/j.progpolymsci.2020.101220.

[ref9] Zhou D.; Shanmukaraj D.; Tkacheva A.; Armand M.; Wang G. Polymer Electrolytes for Lithium-Based Batteries: Advances and Prospects. Chem. 2019, 5, 2326–2352. 10.1016/j.chempr.2019.05.009.

[ref10] Borodin O.; Smith G. D. Mechanism of Ion Transport in Amorphous Poly(ethylene oxide)/LiTFSI from Molecular Dynamics Simulations. Macromolecules 2006, 39, 1620–1629. 10.1021/ma052277v.

[ref11] Maitra A.; Heuer A. Cation Transport in Polymer Electrolytes: A Microscopic Approach. Phys. Rev. Lett. 2007, 98, 227802 10.1103/PhysRevLett.98.227802. 17677880

[ref12] Diddens D.; Heuer A.; Borodin O. Understanding the Lithium Transport within a Rouse-Based Model for a PEO/LiTFSI Polymer Electrolyte. Macromolecules 2010, 43, 2028–2036. 10.1021/ma901893h.

[ref13] Devaux D.; Bouchet R.; Glé D.; Denoyel R. Mechanism of ion transport in PEO/LiTFSI complexes: Effect of temperature, molecular weight and end groups. Solid State Ionics 2012, 227, 119–127. 10.1016/j.ssi.2012.09.020.

[ref14] Brooks D. J.; Merinov B. V.; Goddard W. A. III; Kozinsky B.; Mailoa J. Atomistic Description of Ionic Diffusion in PEO-LiTFSI: Effect of Temperature, Molecular Weight, and Ionic Concentration. Macromolecules 2018, 51, 8987–8995. 10.1021/acs.macromol.8b01753.

[ref15] Sundararaman S.; Halat D. M.; Choo Y.; Snyder R. L.; Abel B. A.; Coates G. W.; Reimer J. A.; Balsara N. P.; Prendergast D. Exploring the Ion Solvation Environments in Solid-State Polymer Electrolytes through Free-Energy Sampling. Macromolecules 2021, 54, 8590–8600. 10.1021/acs.macromol.1c01417.

[ref16] Fong K. D.; Self J.; McCloskey B. D.; Persson K. A. Ion Correlations and Their Impact on Transport in Polymer-Based Electrolytes. Macromolecules 2021, 54, 2575–2591. 10.1021/acs.macromol.0c02545.

[ref17] Gerz I.; Lindh E. M.; Thordarson P.; Edman L.; Kullgren J.; Mindemark J. Oligomer Electrolytes for Light-Emitting Electrochemical Cells: Influence of the End Groups on Ion Coordination, Ion Binding, and Turn-on Kinetics. ACS Appl. Mater. Interfaces 2019, 11, 40372–40381. 10.1021/acsami.9b15233. 31621280

[ref18] Yu D.; Troya D.; Korovich A. G.; Bostwick J. E.; Colby R. H.; Madsen L. A. Uncorrelated Lithium-Ion Hopping in a Dynamic Solvent-Anion Network. ACS Energy Lett. 2023, 8, 1944–1951. 10.1021/acsenergylett.3c00454. 37090169 PMC10112391

[ref19] Gudla H.; Zhang C.; Brandell D. Effects of Solvent Polarity on Li-ion Diffusion in Polymer Electrolytes: An All-Atom Molecular Dynamics Study with Charge Scaling. J. Phys. Chem. B 2020, 124, 8124–8131. 10.1021/acs.jpcb.0c05108. 32840375 PMC7503542

[ref20] Fan J.; Marzke R. F.; Angeill C. A. Conductivity vs NMR Correlation Times, And Decoupled Cation Motion in Polymer-In-Salt Electrolytes. MRS Proc. 1992, 293, 87–92. 10.1557/PROC-293-87.

[ref21] Mishra R.; Baskaran N.; Ramakrishnan P. A.; Rao K. J. Lithium ion conduction in extreme polymer in salt regime. Solid State Ionics 1998, 112, 261–273. 10.1016/S0167-2738(98)00209-4.

[ref22] Borodin O.; Smith G. D.; Henderson W. Li+ Cation Environment, Transport, and Mechanical Properties of the LiTFSI Doped N-Methyl-N-alkylpyrrolidinium+TFSI- Ionic Liquids. J. Phys. Chem. B 2006, 110, 16879–16886. 10.1021/jp061930t. 16927976

[ref23] Smith G. D.; Borodin O. Lithium Battery Electrolyte Stability and Performance from Molecular Modeling and Simulations. In Batteries for Sustainability: Selected Entries from the Encyclopedia of Sustainability Science and Technology; Brodd R. J., Ed.; Springer: New York, 2013; pp 195–237.

[ref24] Rosenwinkel M. P.; Schönhoff M. Lithium Transference Numbers in PEO/LiTFSA Electrolytes Determined by Electrophoretic NMR. J. Electrochem. Soc. 2019, 166, A1977 10.1149/2.0831910jes.

[ref25] Rosenwinkel M. P.; Andersson R.; Mindemark J.; Schönhoff M. Coordination Effects in Polymer Electrolytes: Fast Li+ Transport by Weak Ion Binding. J. Phys. Chem. C 2020, 124, 23588–23596. 10.1021/acs.jpcc.0c08369.

[ref26] Fonseca C. P.; Rosa D. S.; Gaboardi F.; Neves S. Development of a biodegradable polymer electrolyte for rechargeable batteries. J. Power Sources 2006, 155, 381–384. 10.1016/j.jpowsour.2005.05.004.

[ref27] Mindemark J.; Sun B.; Törmä E.; Brandell D. High-performance solid polymer electrolytes for lithium batteries operational at ambient temperature. J. Power Sources 2015, 298, 166–170. 10.1016/j.jpowsour.2015.08.035.

[ref28] Sun B.; Mindemark J.; Morozov E. V.; Costa L. T.; Bergman M.; Johansson P.; Fang Y.; Furó I.; Brandell D. Ion transport in polycarbonate based solid polymer electrolytes: experimental and computational investigations. Phys. Chem. Chem. Phys. 2016, 18, 9504–9513. 10.1039/C6CP00757K. 26984668

[ref29] Molinari N.; Mailoa J. P.; Kozinsky B. Effect of Salt Concentration on Ion Clustering and Transport in Polymer Solid Electrolytes: A Molecular Dynamics Study of PEO-LiTFSI. Chem. Mater. 2018, 30, 6298–6306. 10.1021/acs.chemmater.8b01955.

[ref30] Martínez L.; Andrade R.; Birgin E. G.; Martínez J. M. PACKMOL: A package for building initial configurations for molecular dynamics simulations. J. Comput. Chem. 2009, 30, 2157–2164. 10.1002/jcc.21224. 19229944

[ref31] Abraham M. J.; Murtola T.; Schulz R.; Páll S.; Smith J. C.; Hess B.; Lindahl E. GROMACS: High performance molecular simulations through multi-level parallelism from laptops to supercomputers. SoftwareX 2015, 1–2, 19–25. 10.1016/j.softx.2015.06.001.

[ref32] Berendsen H. J. C.; Postma J. P. M.; van Gunsteren W. F.; DiNola A.; Haak J. R. Molecular dynamics with coupling to an external bath. J. Chem. Phys. 1984, 81, 3684–3690. 10.1063/1.448118.

[ref33] Parrinello M.; Rahman A. Polymorphic transitions in single crystals: A new molecular dynamics method. J. Appl. Phys. 1981, 52, 7182–7190. 10.1063/1.328693.

[ref34] Eriksson T.; Mace A.; Mindemark J.; Brandell D. The role of coordination strength in solid polymer electrolytes: compositional dependence of transference numbers in the poly(ε-caprolactone)-poly(trimethylene carbonate) system. Phys. Chem. Chem. Phys. 2021, 23, 25550–25557. 10.1039/D1CP03929F. 34781333 PMC8612359

[ref35] Mao G.; Saboungi M.-L.; Price D. L.; Armand M. B.; Howells W. S. Structure of Liquid PEO-LiTFSI Electrolyte. Phys. Rev. Lett. 2000, 84, 5536–5539. 10.1103/PhysRevLett.84.5536. 10990988

[ref36] Kameda Y.; Umebayashi Y.; Takeuchi M.; Wahab M. A.; Fukuda S.; Ishiguro S.-i.; Sasaki M.; Amo Y.; Usuki T. Solvation Structure of Li+ in Concentrated LiPF6-Propylene Carbonate Solutions. J. Phys. Chem. B 2007, 111, 6104–6109. 10.1021/jp072597b. 17497919

[ref37] Liu H.; Luo X.; Sokolov A. P.; Paddison S. J. Quantitative Evidence of Mobile Ion Hopping in Polymerized Ionic Liquids. J. Phys. Chem. B 2021, 125, 372–381. 10.1021/acs.jpcb.0c06916. 33393762

[ref38] Mogurampelly S.; Keith J. R.; Ganesan V. Mechanisms Underlying Ion Transport in Polymerized Ionic Liquids. J. Am. Chem. Soc. 2017, 139, 9511–9514. 10.1021/jacs.7b05579. 28686437

[ref39] McDaniel J. G.; Son C. Y. Ion Correlation and Collective Dynamics in BMIM/BF4-Based Organic Electrolytes: From Dilute Solutions to the Ionic Liquid Limit. J. Phys. Chem. B 2018, 122, 7154–7169. 10.1021/acs.jpcb.8b04886. 29927596

[ref40] Zhang Y.; Maginn E. J. Direct Correlation between Ionic Liquid Transport Properties and Ion Pair Lifetimes: A Molecular Dynamics Study. J. Phys. Chem. Lett. 2015, 6, 700–705. 10.1021/acs.jpclett.5b00003. 26262489

[ref41] Timachova K.; Watanabe H.; Balsara N. P. Effect of Molecular Weight and Salt Concentration on Ion Transport and the Transference Number in Polymer Electrolytes. Macromolecules 2015, 48, 7882–7888. 10.1021/acs.macromol.5b01724.

[ref42] Andersson R.; Emilsson S.; Hernández G.; Johansson M.; Mindemark J. Influence of Molecular Weight and End Groups on Ion Transport in Weakly and Strongly Coordinating Polymer Electrolytes. ChemElectroChem 2024, 11, e202400415 10.1002/celc.202400415.

[ref43] Borodin O.; Smith G. D. Li+Transport Mechanism in Oligo(Ethylene Oxide)s Compared to Carbonates. J. Sol. Chem. 2007, 36, 803–813. 10.1007/s10953-007-9146-1.

[ref44] Do C.; Lunkenheimer P.; Diddens D.; Götz M.; Weiß M.; Loidl A.; Sun X.-G.; Allgaier J.; Ohl M. Li+ Transport in Poly(Ethylene Oxide) Based Electrolytes: Neutron Scattering, Dielectric Spectroscopy, and Molecular Dynamics Simulations. Phys. Rev. Lett. 2013, 111, 018301 10.1103/PhysRevLett.111.018301. 23863028

[ref45] Forsyth M.; Jiazeng S.; MacFarlane D. R. Novel high salt content polymer electrolytes based on high Tg polymers. Electrochim. Acta 2000, 45, 1249–1254. 10.1016/S0013-4686(99)00328-X.

