# Supplementary material for: To Hop or Not to Hop: Unveiling Different Modes of Ion Transport in Solid Polymer Electrolytes through Molecular Dynamics Simulations
Source: ACS Appl Polym Mater. 2025 Apr 15;7(8):4716–24. doi: 10.1021/acsapm.4c03724 (PMC12038787; doi:10.1021/acsapm.4c03724)
Supplement: Supplementary file 1 — ap4c03724_si_001.pdf [file ap4c03724_si_001.pdf]

Supporting Information for:

# To hop or not to hop – unveiling different modes of ion transport in solid polymer electrolytes through molecular dynamics simulations

Harish Gudla[1], Anne Hockmann[2], Daniel Brandell[1], Jonas Mindemark[1*]
[1]Department of Chemistry − Ångström Laboratory, Uppsala University, Box 538, SE-751 21 Uppsala, Sweden
[2]Institute of Physical Chemistry, University of Münster, Corrensstr. 28/30, 48149 Münster, Germany

[*]Corresponding author. E-mail: jonas.mindemark@kemi.uu.se

S1. Simulation details ........ S2
S2. Glass transition temperature ($T_g$) ........ S2
S3. Radial distribution functions and coordination number functions ........ S3
S4. Ion transport mechanisms ........ S4
S5. Mean residence time calculation ........ S6
S6. Mean-square displacement ........ S6
S7. Probabilities of transport mechanisms ........ S7
S8. End group dependence ........ S8
S9. References ........ S9

## S1. Simulation details

Table S1. Left, polymer chain lengths, expressed as the degree of polymerization ($n$), molecular weights $M_n$ (g mol$^{-1}$), and calculated $T_g$ (error values in parentheses) for the simulated polymer systems. Right, salt concentration ($x$) and the number of ions in the simulation box.

| **Chain length (n)** | **$M_n$ (g mol$^{-1}$)** | **$T_g$ (K)** | **$x$ = [Li$^+$]/[monomer]** | **No. of ions** |
|---|---|---|---|---|
| | PEO | | | |
| 4 | 238.28 | 306 (9) | LiTFSI | |
| 25 | 1119.33 | 310 (12) | 0 | 0 |
| 250 | 10987.12 | 314 (10) | 0.08 | 80 |
| | PCL | | 0.7 | 700 |
| 4 | 532.67 | 303 (11) | 1.0 | 1000 |
| 24 | 2815.52 | 306 (9) | | |
| 100 | 11490.36 | 309 (11) | | |

## S2. Glass transition temperature ($T_g$)

The $T_g$ of a polymer system can be determined from the change in the density values as a function of temperature.

$$\rho_g(T) = \rho_r(T)|_{T=T_g} \quad \text{(S1)}$$

The temperature dependence of density for polymer in the glass state $\rho_g(T)$ (low temperature region) differs from that for polymer in rubber state $\rho_r(T)$ (high temperature region). The tangents of these two functions intersect at one temperature, which gives the $T_g$.[1]

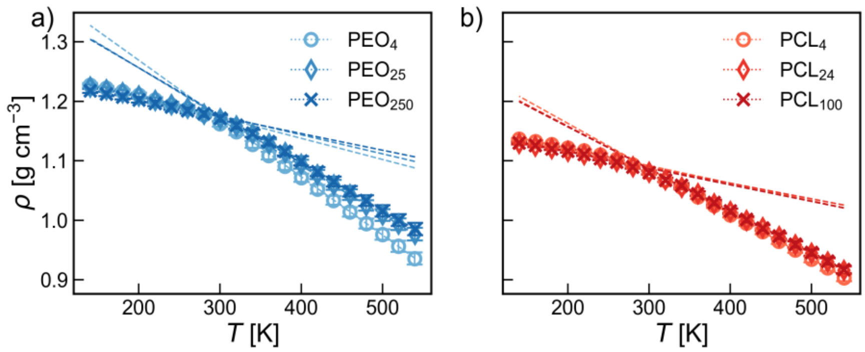


Figure S1. Densities ($\rho$) of the polymer PEO (a) and PCL (b) as a function of temperature ($T$) to determine the glass transition temperature ($T_g$) at different molecular weights.

## S3. Radial distribution functions and coordination number functions

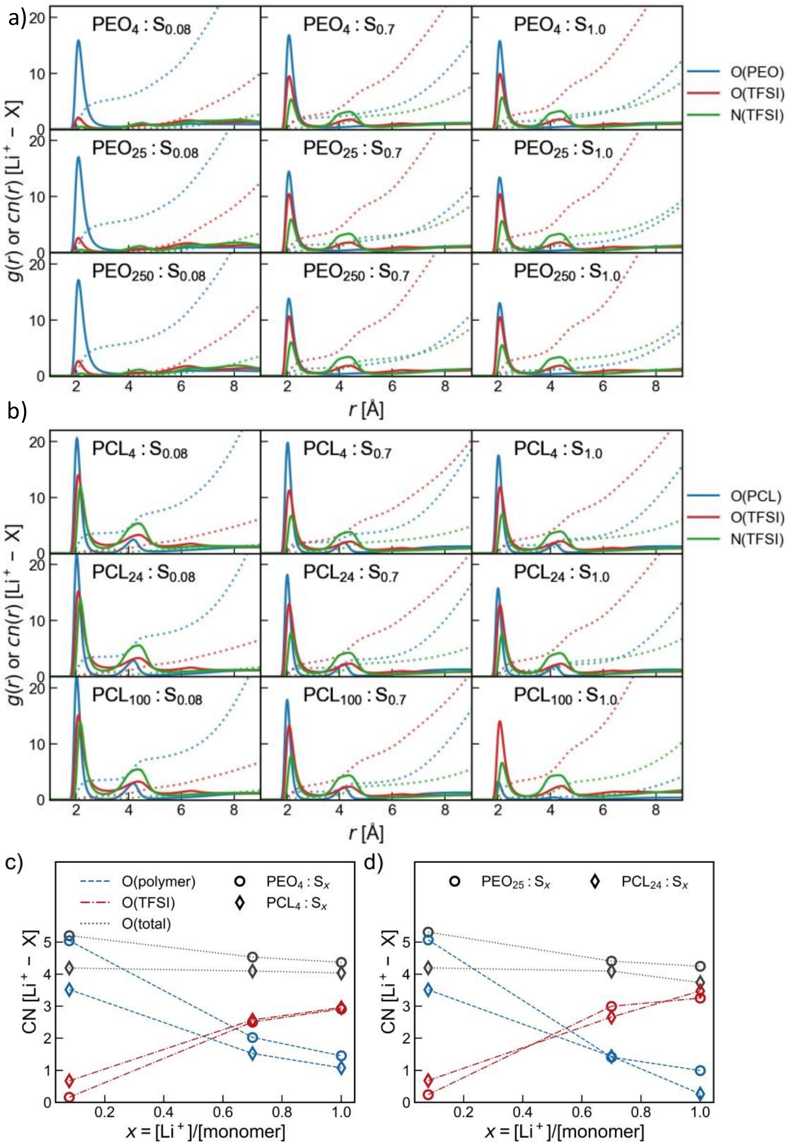


Figure S2. The radial distribution functions ($g(r)$; solid) and the coordination number functions (cn($r$); dashed) for PEO:LiTFSI systems (a) and PCL:LiTFSI systems (b) at different molecular weights and concentrations. The coordination numbers (CN) of $Li^+$–O(polymer), $Li^+$–O(TFSI) and $Li^+$–O(total) in the first coordination shell (first minimum in $g(r)$) as function of salt concentration for $PEO_4$ and $PCL_4$ systems (c) and for $PEO_{25}$ and $PCL_{24}$ systems (d).

## S4. Ion transport mechanisms

The method used in this work to observe the changes in the coordination is based on previous MD studies on polymer electrolytes[2] and polymerized ionic liquids[3,4], but has here been extended to include coordination changes of both anions and polymers together with changes in the total coordination number.

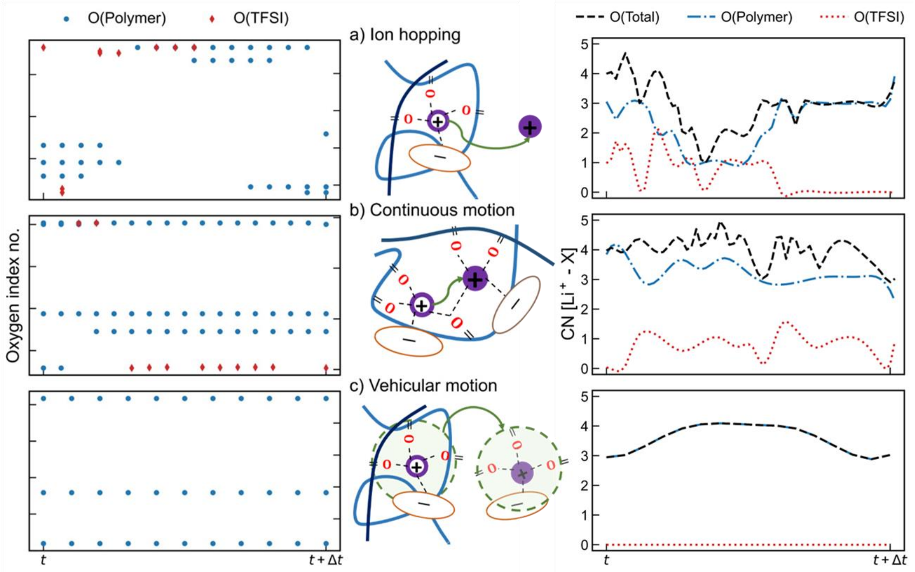


Figure S3. Time evolution plots of O(polymer) and O(TFSI) index numbers (left) and total coordination number (CN [$Li^+$–O(total)]) (right) of a random $Li^+$ in the system $PCL_n$:$S_{0.08}$ from $t$ to $t + \Delta t$ and visualizing (centre) the characterization of types of ion transport mechanisms a) ion hopping event b) continuous motion and c) vehicular motion. Colors: blue and dark blue lines – different polymer chains; red – O atoms; purple – cation; orange and brown ovals – different anions; green arrow – motion of cation.

Each O atom in the simulation box has a distinct index number and details of which specific polymer or anion it belongs to. Therefore, at each time step, the oxygen atoms coordinating to each $Li^+$ were investigated and assigned to the polymer or the anion, respectively. In detail, three lists were created, a polymer list with all the O index numbers belonging to the polymer, a corresponding TFSI list including all the O index numbers of the anions, and finally a CN list with the total number of coordinating O atoms between $t$ and $t + \Delta t$. Thus, after this step, two lists with O index numbers and one list with CNs were obtained for each cation at every time step of the simulation. Thereafter, these three lists for each $Li^+$ at time $t$ were compared to the lists of the same cation at $t + \Delta t$. This comparison enables the three distinct transport modes to be captured, as illustrated in Figure S3. These mechanisms are defined as follows:

1. Ion hopping (Hop): The O index number list at $t + \Delta t$ is completely changed from the list at $t$, and during this time $\Delta t$, the total CN is either 0 or 1 (see Figure S3a; right-side plot near $t + \Delta t$), *i.e.*, at this time point the $Li^+$ is barely coordinating with either anion or polymer. Here, the cation has basically changed its whole coordination environment, and there is a time point when the cation is completely or almost free, and an independent hop occurs between two different coordination environments.
2. Continuous motion (Cont.): The O index number lists at $t$ and $t + \Delta t$ have a minimum change of one O atoms and maximum of one less than total coordinated O atoms, and only small fluctuation in total CN (see Figure S3b) within this time frame, *i.e.*, the coordination shell size is almost constant. Here, the cation has partially changed its coordination environment, *i.e.*, it is moving along or between the polymer chain or anions.
3. Vehicular motion (Vehi.): There is no change in the O index number lists at $t$ and $t + \Delta t$, and during this time $\Delta t$ a single coordination site could vary. The fluctuation in the total CNs (see Figure S3c)

is either 0 or 1 *i.e.*, the coordination shell size is almost constant. Here, the cation moves together with the same coordination environment.

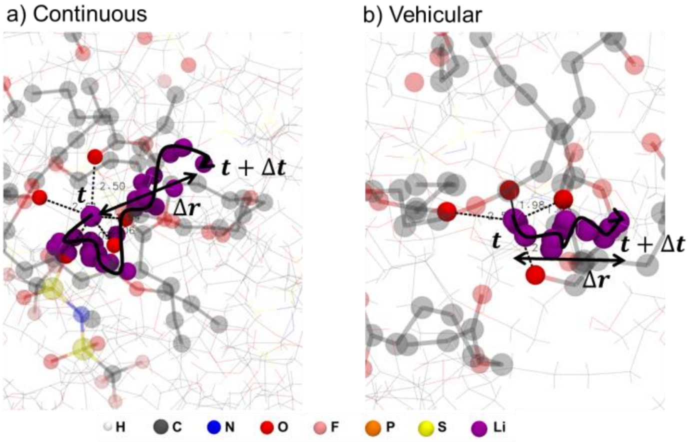


Figure S4. Representative snapshots for a) continuous and b) vehicular transport, along with the time evolution of $Li^+$ (purple) from $t$ to $t + \Delta t$ and travelled distance of $\Delta r$ is shown at different timesteps.

The percentage of effective events as a function of cut-off distance ($\Delta r$) and time step ($\Delta t$) are shown in Figure S5a,b. To estimate the anion dynamics in the polymer-mediated continuous motion, the percentage of events where the coordinating anion group changes within a time step $\Delta t$ was calculated and plotted in Figure S5c for the low salt concentration systems ($x$ = 0.08). A similar analysis was performed to estimate the polymer dynamics in the anion-mediated continuous motion and plotted in Figure S5d for the higher salt concentration systems ($x$ = 0.7, 1.0).

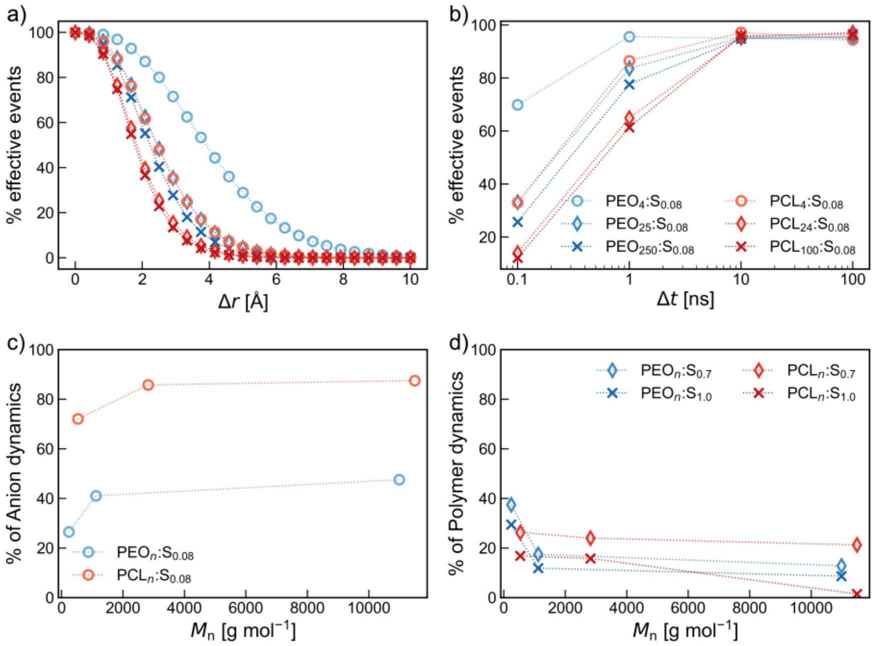


Figure S5. a) The percentage of effective events as a function of $\Delta r$ (distance travelled by $Li^+$ in time step $\Delta t$ = 0.1 ns) and b) the percentage of effective events as a function of $\Delta t$ with a cut-off distance $\Delta r$ of 3 Å for the systems $PEO_n$:$S_{0.08}$ and $PCL_n$:$S_{0.08}$. c) The percentage of anion dynamics involved in polymer-mediated continuous motion as function of molecular weight for PEO and PCL at $x$ = 0.08. d) The percentage of polymer dynamics involved in anion-mediated continuous motion as function of molecular weight for PEO and PCL at $x$ = 0.7 and 1.0.

## S5. Mean residence time calculation

The residence times of $Li^+$–O(polymer) and $Li^+$–O(TFSI) were calculated from their contact correlation function $C(t)$ using the reactive flux method:[5,6]

$$C(t) = \frac{\langle h(t_0)h(t_0+t)\rangle}{\langle h(t_0)\rangle} \tag{S2}$$

where $h$(t) is 1 if $Li^+$ – O(X) were in contact cut-off distance and is 0 otherwise. Here the first minima in $g(r)$ of $Li^+$ – O(polymer) and $Li^+$ – O(TFSI) are considered as cut-off distance. $C(t)$ is then fitted to a biexponential decay function and the residence time $\tau$ is estimated as

$$\tau = \int_0^\infty C(t)\, dt \tag{S3}$$

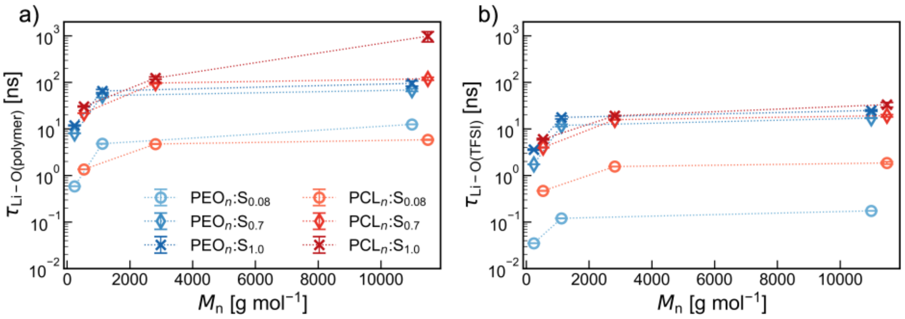


Figure S6. The average residence times of (a) $Li^+$–O(polymer) $\tau_{\text{Li-O(polymer)}}$ and (b) $Li^+$–O(TFSI) $\tau_{\text{Li-O(TFSI)}}$ as a function of polymer molecular weight for both polymers at differenct salt concentrations.

## S6. Mean-square displacement

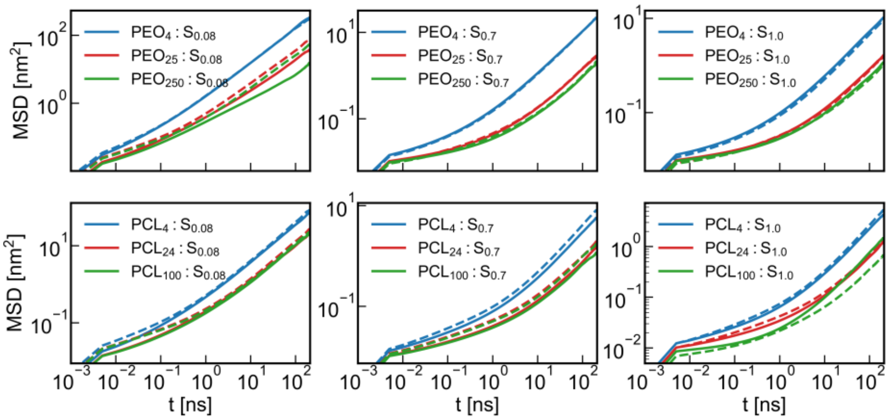


Figure S7. The mean-square displacements (MSD) of $Li^+$ (solid) and N(TFSI) (dashed) of polymer for PEO:LiTFSI (top) and PCL:LiTFSI (bottom) as function of simulation time for different molecular weights and concentrations.

## S7. Probabilities of transport mechanisms

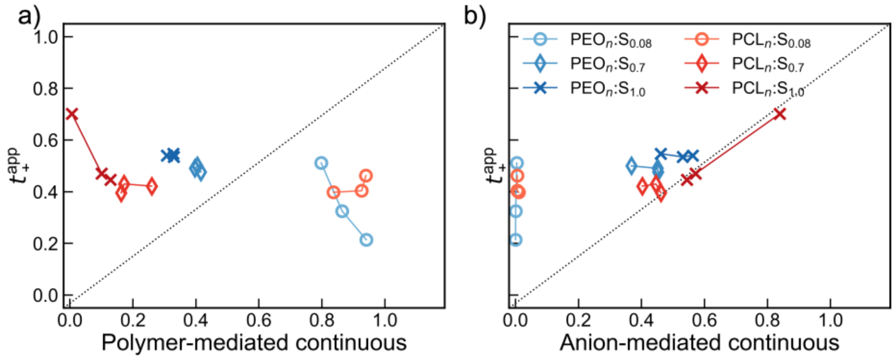


Figure S8. Correlation plots between cation transference number and probabilities of polymer-mediated continuous motion (a) and anion-mediated continuous motion (b) for PEO:LiTFSI and PCL:LiTFSI systems.

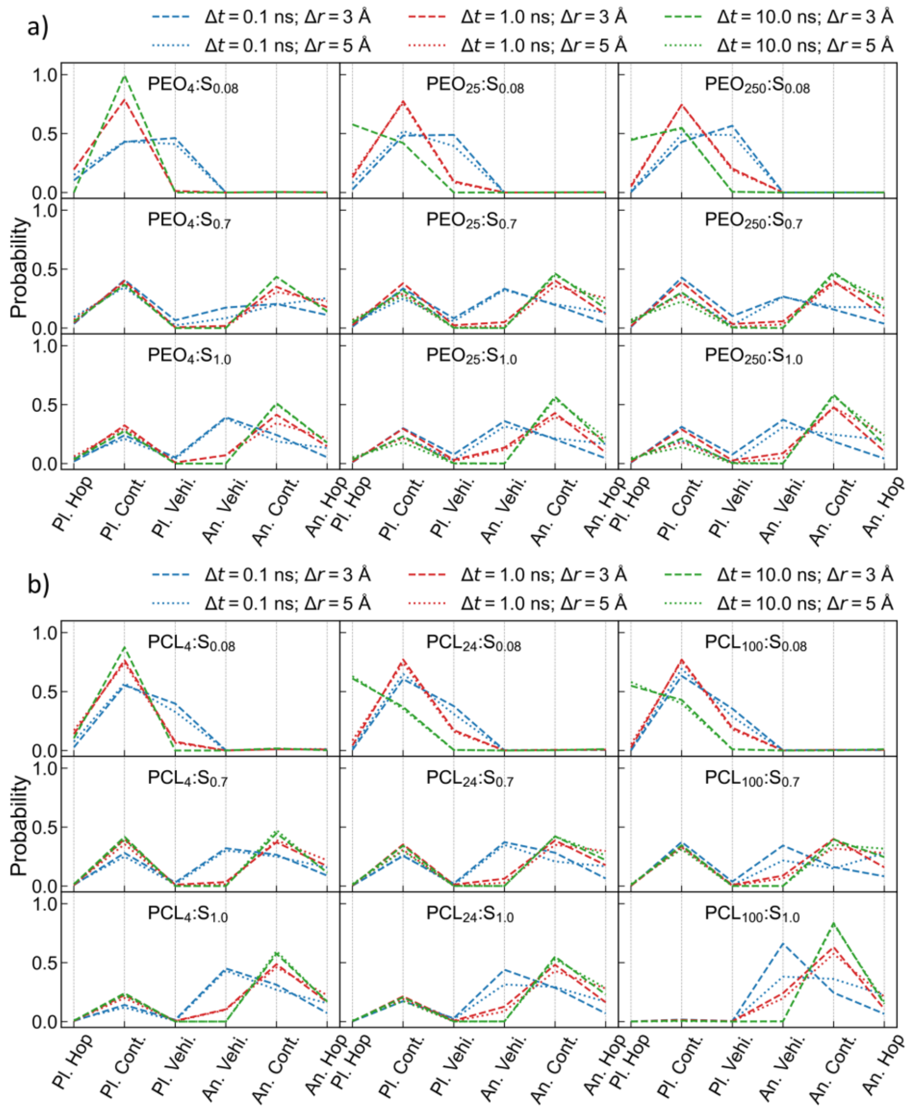


Figure S9. Probability of each transport mechanisms for all PEO:LiTFSI systems (a) and PCL:LiTFSI systems (b) at different Δ*r* and Δ*t*.

## S8. End group dependence

To estimate the effect of end group on the transport properties and mechanisms, the -OH end group is replaced with $-CH_3$. The new simulations are only performed for the polymer systems with smallest molecular weights i.e., $PEO_4$:$S_x$ and $PCL_4$:$S_x$ as they will have the highest number of end groups.

In Figure S10, probability of type of ion transport mechanisms for the -OH (solid) and $-CH_3$ (dashed) end group at different $\Delta r$ and $\Delta t$ are compared. For both the polymer systems, transport modes are not affected by change in end group, however at higher $\Delta t$'s (10 and 100 ns), transport mode is completely shifted to ion hopping for $-CH_3$ end group and for -OH end group continuous transport is still dominant.

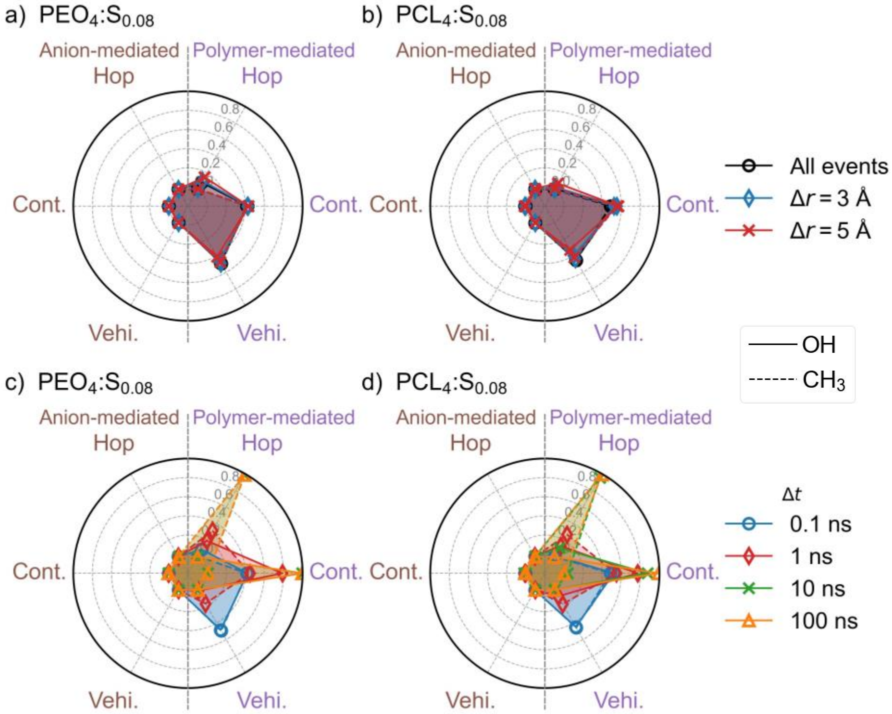


Figure S10. Probability of type of ion transport mechanisms for the -OH (solid) and $-CH_3$ (dashed) end group systems $PEO_4$:$S_{0.08}$ (a,c) and $PCL_4S_{0.08}$ (b,d) where either all (essentially $\Delta r = 0$) or effective events with $\Delta r = 3$ or 5 Å are considered (a,b). Probability of type of ion transport mechanisms at different time steps ($\Delta t$) with $\Delta r = 3$ Å (c,d).

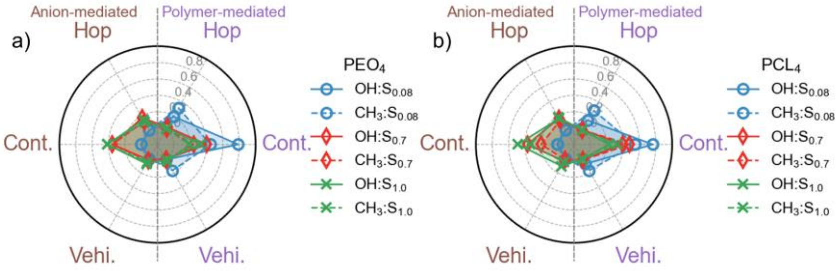


Figure S11. Probability of type of ion transport mechanisms and $Li^+$ transference number at different end groups -OH (solid), $-CH_3$ (dashed), different salt concentrations for both the polymers $PEO_4$ (a) and $PCL_4$ (b) with time step $\Delta t = 1$ ns and effective distance criterion $\Delta r = 3$ Å. The mechanisms are further categorized into the polymer- (purple; right) and anion-mediated (brown; left) ion transport.

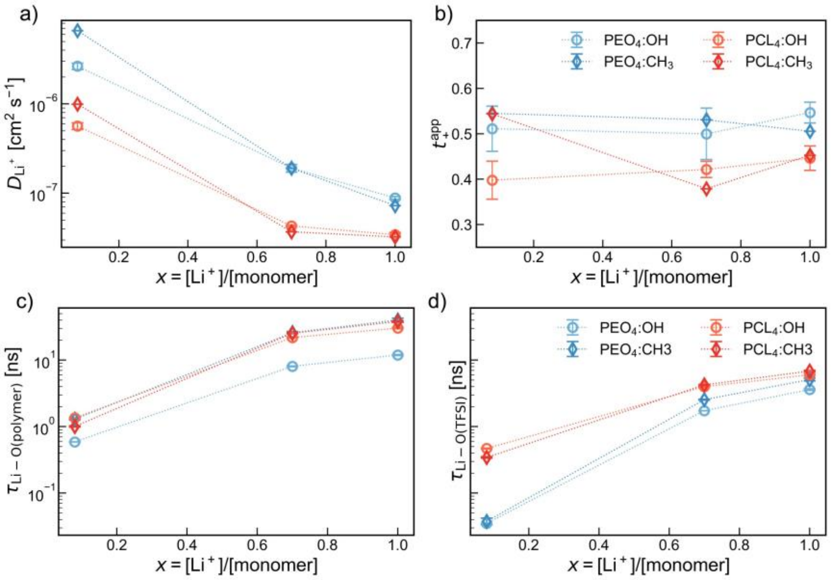


Figure S12. a) The Li+ self-diffusion coefficients ($D_{\mathrm{Li}^+}$), b) apparent cation transference number ($t_+^{\mathrm{app}}$), the average residence times of Li+–O(polymer) $\tau_{\mathrm{Li\text{-}O(polymer)}}$ (c) and Li+–O(TFSI) $\tau_{\mathrm{Li\text{-}O(TFSI)}}$ (d) as a function of salt concentrations for at different end groups -OH (circle), $-CH_3$ (diamond) for both the polymers $PEO_4$ and $PCL_4$.

The probabilities of transport modes in Figure S11 shows that the different end group systems do not affect the dominant transport mechanism even when changing the salt concentration. However, for $S_{0.08}$ systems there is a decrease in continuous motion which was then compensated by an increase in ion hopping and vehicular transport. From Figure S12a,b, a slight increase in transport properties ($D_{\mathrm{Li}^+}$ and $t_+^{\mathrm{app}}$ ) at the same concentration can also be observed and can be attributed to increase in ion hopping. The residence times of Li+–O(TFSI) (Figure S12d) are also not affected by end groups but an increase in $\tau_{\mathrm{Li\text{-}O(polymer)}}$ is evident in Figure S12c at the lowest concentration which could explain the increase in vehicular transport as ions tend to spend more time with polymer chains with $-CH_3$ end group.

## S9. References


(1) Wu, C. Simulated Glass Transition of Poly(Ethylene Oxide) Bulk and Film: A Comparative Study. *J. Phys. Chem. B* **2011**, *115*, 11044–11052.

(2) Brooks, D. J.; Merinov, B. v.; Goddard, W. A.; Kozinsky, B.; Mailoa, J. Atomistic Description of Ionic Diffusion in PEO–LiTFSI: Effect of Temperature, Molecular Weight, and Ionic Concentration. *Macromolecules* **2018**, *51*, 8987–8995.

(3) Liu, H.; Luo, X.; Sokolov, A. P.; Paddison, S. J. Quantitative Evidence of Mobile Ion Hopping in Polymerized Ionic Liquids. *J. Phys. Chem. B 125*, 372–381.

(4) Mogurampelly, S.; Keith, J. R.; Ganesan, V. Mechanisms Underlying Ion Transport in Polymerized Ionic Liquids. *J. Am. Chem. Soc.* **2017**, *139*, 9511–9514.

(5) Luzar, A. Resolving the Hydrogen Bond Dynamics Conundrum. *J. Chem. Phys.* **2000**, *113*, 10663–10675.

(6) Luzar, A.; Chandler, D. Hydrogen-Bond Kinetics in Liquid Water. *Nature* **1995**, *379*, 55–57.
